# Experimental classical entanglement in a 16 acoustic qubit-analogue

**DOI:** 10.1038/s41598-021-03789-5

**Published:** 2021-12-20

**Authors:** M. Arif Hasan, Keith Runge, Pierre A. Deymier

**Affiliations:** 1 Department of Mechanical Engineering, Wayne State University, Detroit, MI 48202 USA; 2 Department of Materials Science and Engineering, The University of Arizona, Tucson, AZ 85721 USA

**Keywords:** Acoustics, Structural materials

## Abstract

The possibility of achieving and controlling scalable classically entangled, i.e., inseparable, multipartite states, would fundamentally challenge the advantages of quantum systems in harnessing the power of complexity in information science. Here, we investigate experimentally the extent of classical entanglement in a $16$ acoustic qubit-analogue platform. The acoustic qubit-analogue, a.k.a., logical phi-bit, results from the spectral partitioning of the nonlinear acoustic field of externally driven coupled waveguides. Each logical phi-bit is a two-level subsystem characterized by two independently measurable phases. The phi-bits are co-located within the same physical space enabling distance independent interactions. We chose a vector state representation of the $16$-phi-bit system which lies in a ${2}^{16}$-dimensional Hilbert space. The calculation of the entropy of entanglement demonstrates the possibility of achieving inseparability of the vector state and of navigating the corresponding Hilbert space. This work suggests a new direction in harnessing the complexity of classical inseparability in information science.

## Introduction

Classical waves such as electromagnetic or acoustic waves can possess attributes reminiscent of quantum entanglement. Classical entanglement is associated with the occurrence of some mathematical and/or physical features of quantum entanglement in the representation of classical waves. Specifically, the emergence of a multipartite tensor product structure in the state space representation of the wave can lead to the notion of inseparability between different degrees of freedom as an aspect of classical entanglement. Considering two two-level degrees of freedom of the same classical wave, its four-dimensional product Hilbert space can contain superpositions of product states of the wave that are not algebraically separable into a single product. This is the case for spin angular momentum, orbital angular momentum (OAM), polarization, direction of propagation and/or radial degree of freedom in optical beams^1–10^ as well as direction of propagation, normal modes analogous to OAM, and/or pseudospin in acoustic or elastic waveguides^11–14^. In linear classical systems, the dimension of the product Hilbert space is, however, limited by the number of available degrees of freedom. In contrast, nonlinear classical systems may support nonlinear waves that span Hilbert spaces with dimension scaling exponentially^15,16^. The nonlinearity enables wave-wave interaction, necessary to achieve wave mixing, allowing the formation of waves for which the frequency and wave number are the sums of the frequencies and wave numbers of parent linear waves. For example, the mixing of OAM modes in nonlinear interaction by simultaneously exploiting different photonic degrees of freedom has been investigated in optical waves supporting OAM^17,18^. However, the classical forms of entanglement between degrees of freedom of a single system differ from entanglement between degrees of freedom of different subsystems^19^. Nonetheless, these subsystems may be subject to representation since the partitioning of a physical system into subsystems is not unique and conditioned by measurement^20,21^. Classical entanglement may also be distinguished from quantum entanglement in that it does not exhibit quantum nonlocality^22,23^. However, partitioning of classical waves outside the spatial domain can co-locate the subsystems in the same physical space, obviating the issue of distance dependent interactions. Finally, classically entangled superpositions of states involve directly measurable complex coefficients that are amplitudes (with phase) in contrast to quantum superpositions which coefficients are probability amplitudes obeying the Born rule. Consequently, classically entanglement superpositions of states are not susceptible to decoherence.

Large multi-qubit (10 to 25 qubits) entangled states have been demonstrated in experimental systems supporting superconducting or photonic qubits, or trapped ions^24–35^. Notwithstanding distinguishing features between quantum and classical entanglement, the possibility of preparing and controlling exponentially complex nonseparable nonlinear superpositions of acoustic waves may offer an alternative to genuine quantum systems to realize experimentally the advantage of complexity and parallelism of entanglement. We investigate experimentally the extent of classical entanglement in a 16 acoustic qubit-analogue platform. The acoustic qubit-analogues, subsequently called phase-bits or phi-bits, result from the spectral partitioning of the nonlinear acoustic field in an externally driven array of coupled acoustic waveguides. Each phi-bit is a two-level subsystem characterized by two independent measurable phases owing to the degree of freedom across the array of waveguides. A representation of the large multi-phibit system is constructed to demonstrate the possibility of achieving inseparability of the vector state of the multipartite system in a ${2}^{16}$-dimensional Hilbert space. To illustrate classical entanglement and our ability to easily navigate a substantial portion of the large Hilbert space, we make use of the entropy of entanglement of various partitioning of the multipartite phi-bit system.

## Results

### Physical platform to realize acoustic qubit-analogue

**Figure 1:**
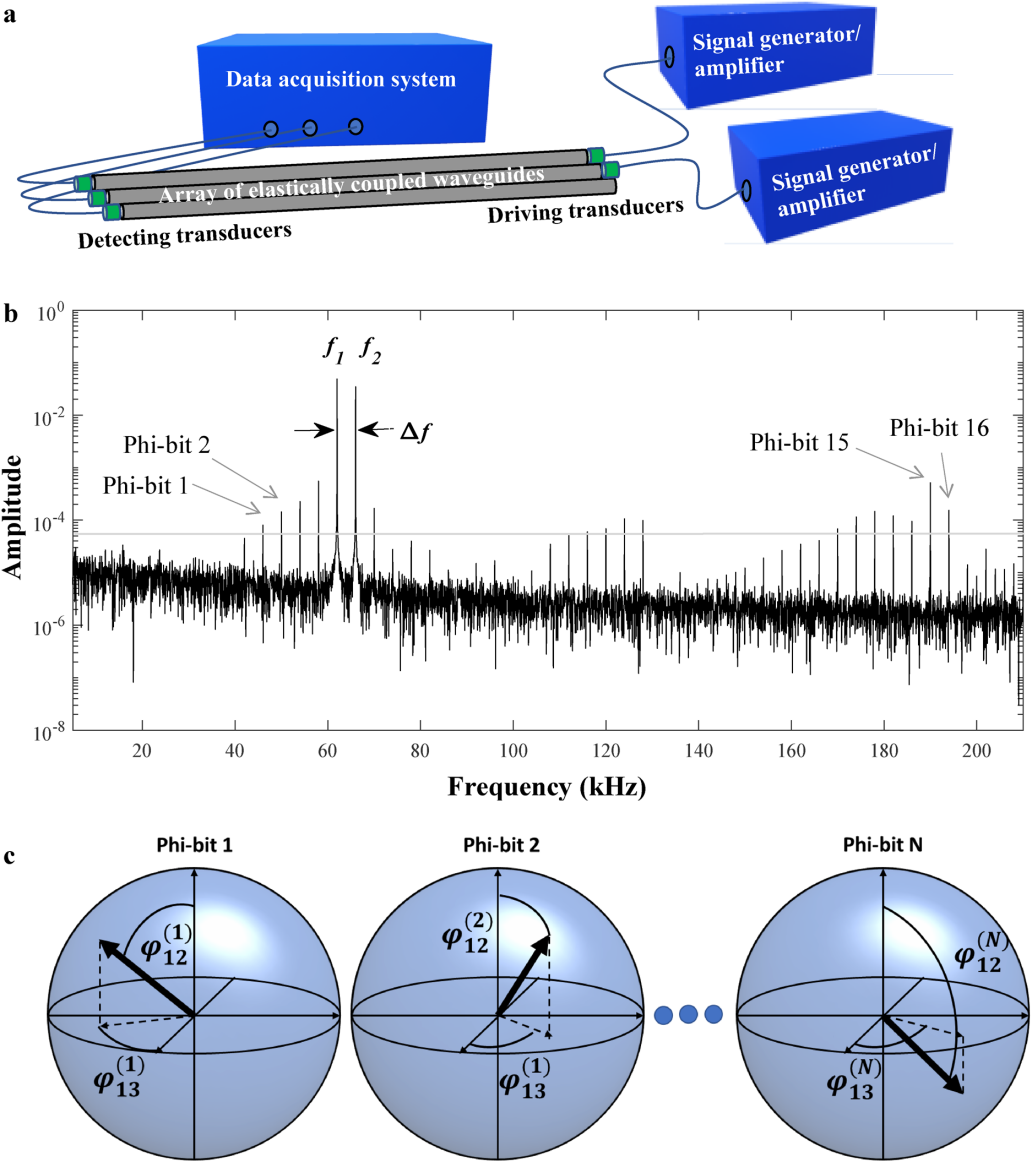
Experimental realization of two-level acoustic qubit-analogue i.e., logical phi-bits. (**a**) Schematic illustration of the experimental nonlinear acoustic waveguide-transducer-amplifier-generator platform. (**b**) Temporal Fourier transform of the rod amplitude, revealing the selection of logical phi-bits. The phi-bits are labelled from the first one to the 16th one in order of increasing frequency. The light grey horizontal line indicates the amplitude threshold for selection of phi-bits. System parameters: ${f}_{1}=62\text{ kHz}$, ${f}_{2}=66\text{ kHz}$, $\Delta f=\left|{f}_{2}-{f}_{1}\right|$. (**c**) Schematic illustration of *N* two-level phi-bit multipartite system.

To physically realize acoustic qubit-analogue i.e., phi-bits, we devise an experimental set up as shown in Fig. 1a (details can be found in the “Methods” section). The one-dimensional coupled acoustic waveguides consist of three aluminum rods arranged in a linear array with a lateral gap filled with epoxy. Transducers drive and detect the acoustic field at the ends of the rods. Function generators and amplifiers excite two driving transducers located on a waveguide at the edge of the array (1) and the middle waveguide (2). The driving waveguides, 1 and 2, are excited with sinusoidal signals with frequency ${f}_{1}$ and ${f}_{2}$. The detecting transducers are connected to an oscilloscope which collects the data. The waveguide, transducer, signal generator, amplifier, acquisition system assembly behaves nonlinearly. The origin of the nonlinearity may be mechanical or electrical, however what follows is independent of that origin. The nonlinearity will lead to a large number of ways of mixing the drivers’ frequencies (a detailed example can be found in the Supplementary Note 1). The measured displacement field at the detection end of the waveguides is the Fourier sum of a large number of linear and nonlinear modes, each with its own characteristic frequency. The frequency of the nonlinear modes results from mixing the drivers’ frequencies. For each nonlinear mode, “*j*” we measure the amplitude of each waveguide, ${C}_{i}^{(j)};i=\text{1,2},3$ and the phase of waveguides 2 and 3 relative to that of waveguide 1, (${\varphi }_{12}^{(j)}$ and ${\varphi }_{13}^{(j)}$). These phases are associated with the degree of freedom across the array of waveguides. Since the partitioning of the Hilbert space of a physical system into subsystems is dictated by the experimental interactions and observables^21^. The elastic field can therefore be interpreted as the superposition of a system of interacting oscillator subsystems with frequencies corresponding to those of the nonlinear modes. Each oscillator system is then identified as a logical phi-bit. Phi-bits are selected in the spectral domain. While selecting logical phi-bits, to eliminate the effects of noise, we put a threshold value of 0.1% of the maximum amplitude of the output rods as shown in Fig. 1b. The phi-bits are selected in the order of increasing frequency (see Fig. 1b).

Each phi-bit is effectively a two-level subsystem characterized by the two independently measurable phase differences between the waveguides, ${\varphi }_{12}^{(j)}$ and ${\varphi }_{13}^{(j)}$. The system composed of three driven and nonlinearly coupled waveguides is therefore represented in the spectral domain by a composite system of *N* (the number of selected nonlinear modes with different mixed frequency) two-level subsystems (Fig. 1c). The states of the subsystems are correlated through the nonlinear interactions of the waveguide-transducer-amplifier-generator assembly.

These correlated states are tunable through control of the components of the driving forces as well as their relative amplitude. Another mechanism of tuning the phi-bit states is via the driving frequencies ${f}_{1}$ and ${f}_{2}$. For instance, in this study, we have chosen to vary the driving frequencies in the form ${f}_{1}-\Delta \nu$ and ${f}_{2}+\Delta \nu$ where $\Delta \nu$ is the detuning parameter.

Since each phi-bit “*j*” is a subsystem with state identifiers ${\varphi }_{12}^{(j)}$ and ${\varphi }_{13}^{(j)}$, we construct a representation of a *N* phi-bit system, (see Supplementary Note 2) that is characterized by a state vector, $\psi$, that lies in a ${2}^{N}$ dimensional Hilbert space. The state vector components are given by $1+{e}^{i{\sum }_{j=1}^{N}{\varphi }_{1{q}_{j}}^{(j)}}$ where ${q}_{j}$ can take on the values 2 or 3. This multipartite tensor product structure is conditioned by the measurability of the phases of each phi-bit. Note that this is but one possible representation of the nonlinear elastic states of the coupled waveguide system. Since the notion of classical entanglement (i.e., nonseparability) is relative to a choice of representation, we have chosen a representation which leads to multipartite states that are likely to be nonseparable. This representation may then be possibly used for nonseparability-based information processing. The complex coefficients of the ${2}^{N}$ states of *N* phi-bits are,

$$   \begin{array}{*{20}c}    \begin{gathered}   \psi _{{00 \ldots 0}}  = \left[ {1 + e^{{i\left( {\varphi _{{12}}^{{\left( 1 \right)}}  + \varphi _{{12}}^{{\left( 2 \right)}}  +  \cdots  + \varphi _{{12}}^{{\left( N \right)}} } \right)}} } \right]/\psi _{{Norm}}  \hfill \\    \hfill \\  \end{gathered}   \\    {\psi _{{00 \ldots 1}}  = \left[ {1 + e^{{i\left( {\varphi _{{12}}^{{\left( 1 \right)}}  + \varphi _{{12}}^{{\left( 2 \right)}}  +  \cdots  + \varphi _{{13}}^{{\left( N \right)}} } \right)}} } \right]/\psi _{{Norm}} }  \\     \vdots   \\    {\psi _{{11 \ldots 1}}  = \left[ {1 + e^{{i\left( {\varphi _{{13}}^{{\left( 1 \right)}}  + \varphi _{{13}}^{{\left( 2 \right)}}  +  \cdots  + \varphi _{{13}}^{{\left( N \right)}} } \right)}} } \right]/\psi _{{Norm}} }  \\   \end{array},  $$

where $${\psi }_{Norm}=\sqrt{{\left[1+{e}^{i\left({\varphi }_{12}^{(1)}+{\varphi }_{12}^{(2)}+\dots +{\varphi }_{12}^{(N)}\right)}\right]}^{2}+\dots +{\left[1+{e}^{i\left({\varphi }_{13}^{(1)}+{\varphi }_{13}^{(2)}+\dots +{\varphi }_{13}^{(N)}\right)}\right]}^{2}}.$$

The normalized components of $$\psi $$ form the complex coefficients of a linear combination of $${2}^{N}$$ dimensional tensor product basis vectors. Let us denote by $$\left|00\dots 0\rangle ,\left|00\dots 1\rangle ,\dots ,\left|11\dots 1\rangle \right.\right.\right.$$ these basis vectors. For each phi-bit “*j*”, the phase differences $${\varphi }_{12}^{(j)}$$ and $${\varphi }_{13}^{(j)}$$ are expressible in terms of good “quantum” numbers, namely the three “*n*” labeling the Eigen vectors $$ \overset{\lower0.5em\hbox{$\smash{\scriptscriptstyle\rightharpoonup}$}}{E} _{n}  $$ representing the modes of vibration across the waveguides and the associated discrete sets of wave numbers, $${k}_{n}$$ representing the vibrations along the waveguides through plane waves (see Supplementary Note 1). Any representation of the elastic state of the system, $$\psi $$, that is a function of the $${\varphi }_{12}^{(j)}$$ and $${\varphi }_{13}^{(j)}$$ is therefore expressible in terms of these “quantum” numbers. For a given representation, such as the one introduced here, there will exist a tensor product structure and tensor product basis in which it can be expanded which is also expressible in terms of $$ \overset{\lower0.5em\hbox{$\smash{\scriptscriptstyle\rightharpoonup}$}}{E} _{n}  $$ and $${k}_{n}$$ (see Supplementary Note 2).

### Classical entanglement in $N$-phi-bits

The displacement field of the nonlinearly coupled waveguides platform is a superposition of states in the ${2}^{N}$ dimensional Hilbert subspace (see Supplementary Note 2). The separability and nonseparability of these superposition of states rely upon the tensor product structure of the Hilbert space associated to the states of the system. We will expand this concept beginning with two logical phi-bits, then three, four and finally all sixteen. The extracted frequency values of the logical phi-bits are listed in Table 1.

**Table 1:** List of logical phi-bits, as identified in Fig. 1b.

Phi-bit	Frequency	Minimum entropy of entanglement	Maximum entropy of entanglement
1	${f}_{1}-4\Delta f$	0.19 (0 kHz)	0.9995 (2.4 kHz)
2	${f}_{1}-3\Delta f$	0.005 (1.4 kHz)	0.976 (0.4 kHz)
3	${f}_{1}-2\Delta f$	0.008 (0.4 kHz)	0.9966 (2.2 kHz)
4	${f}_{1}-\Delta f$	0.24 (0 kHz)	0.87 (1.2 kHz)
5	${f}_{2}+\Delta f$	0.001 (2.6 kHz)	0.3 (0.4 kHz)
6	${f}_{1}+{f}_{2}-3\Delta f$	0.005 (1.8 kHz)	0.994 (3.6 kHz)
7	${f}_{1}+{f}_{2}-2\Delta f$	0.16 (4 kHz)	0.99 (1.4 kHz)
8	${f}_{1}+{f}_{2}-\Delta f$	0.11 (0.4 kHz)	0.995 (0.2 kHz)
9	${f}_{1}+{f}_{2}$	0.58 (0 kHz)	0.943 (1.2 kHz)
10	$2{f}_{1}+{f}_{2}-5\Delta f$	0.007 (1.4 kHz)	1 (0.2 kHz)
11	$2{f}_{1}+{f}_{2}-4\Delta f$	0.007 (1.8 kHz)	1 (0.2 kHz)
12	$2{f}_{1}+{f}_{2}-3\Delta f$	0.001 (3.6 kHz)	1 (3.2 kHz)
13	$2{f}_{1}+{f}_{2}-2\Delta f$	0.003 (1 kHz)	1 (4 kHz)
14	$2{f}_{1}+{f}_{2}-\Delta f$	0.005 (0.2 kHz)	1 (3 kHz)
15	$2{f}_{1}+{f}_{2}$	0.007 (1.2 kHz)	0.75 (0 kHz)
16	$2{f}_{1}+{f}_{2}+\Delta f$	0.002 (2.6 kHz)	0.594 (1 kHz)

The phi-bits are listed in the order of increasing frequencies. Columns 3 and 4 list the minimum and maximum entanglement entropy values for single phi-bit traced state. Detuning frequencies corresponding to these maxima and minima are shown parenthetically.

#### ${{N}}=2$

This bipartite system consists of only two logical phi-bits (phi-bits 9 and 15). In this simple case, we can write analytically the condition for separability of the state in the form:

$${\psi }_{01}{\psi }_{10}={\psi }_{00}{\psi }_{11},$$

where $${\psi }_{00}=\left[1+{e}^{i\left({\varphi }_{12}^{(9)}+{\varphi }_{12}^{(15)}\right)}\right]/{\psi }_{Norm}$$, $${\psi }_{01}=\left[1+{e}^{i\left({\varphi }_{12}^{(9)}+{\varphi }_{13}^{(15)}\right)}\right]/{\psi }_{Norm}$$, $${\psi }_{10}=\left[1+{e}^{i\left({\varphi }_{13}^{(9)}+{\varphi }_{12}^{(15)}\right)}\right]/{\psi }_{Norm}$$, $${\psi }_{11}=\left[1+{e}^{i\left({\varphi }_{13}^{(9)}+{\varphi }_{13}^{(15)}\right)}\right]/{\psi }_{Norm}$$, and $${\psi }_{Norm}=\sqrt{{\left[1+{e}^{i\left({\varphi }_{12}^{(9)}+{\varphi }_{12}^{(15)}\right)}\right]}^{2}+{\left[1+{e}^{i\left({\varphi }_{12}^{(9)}+{\varphi }_{13}^{(15)}\right)}\right]}^{2}+{\left[1+{e}^{i\left({\varphi }_{13}^{(9)}+{\varphi }_{12}^{(15)}\right)}\right]}^{2}+{\left[1+{e}^{i\left({\varphi }_{13}^{(9)}+{\varphi }_{13}^{(15)}\right)}\right]}^{2}}$$.

**Figure 2:**
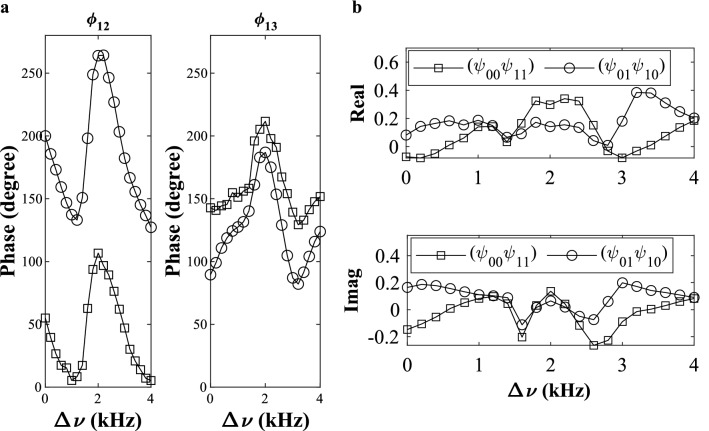
Nonseparability of $N=2$ phi-bits. (**a**) Experimental phase differences between the coupled waveguides (${\varphi }_{12}$ and ${\varphi }_{13}$) for the two logical phi-bits (9, 15). In the plot, open circle corresponds to ${\varphi }_{12}^{(9)}$ and ${\varphi }_{13}^{(9)}$ and open square corresponds to ${\varphi }_{12}^{(15)}$ and ${\varphi }_{13}^{(15)}$. (**b**) Real and imaginary components of ${\psi }_{01}{\psi }_{10}$ and ${\psi }_{00}{\psi }_{11}$.

**Figure 3:**
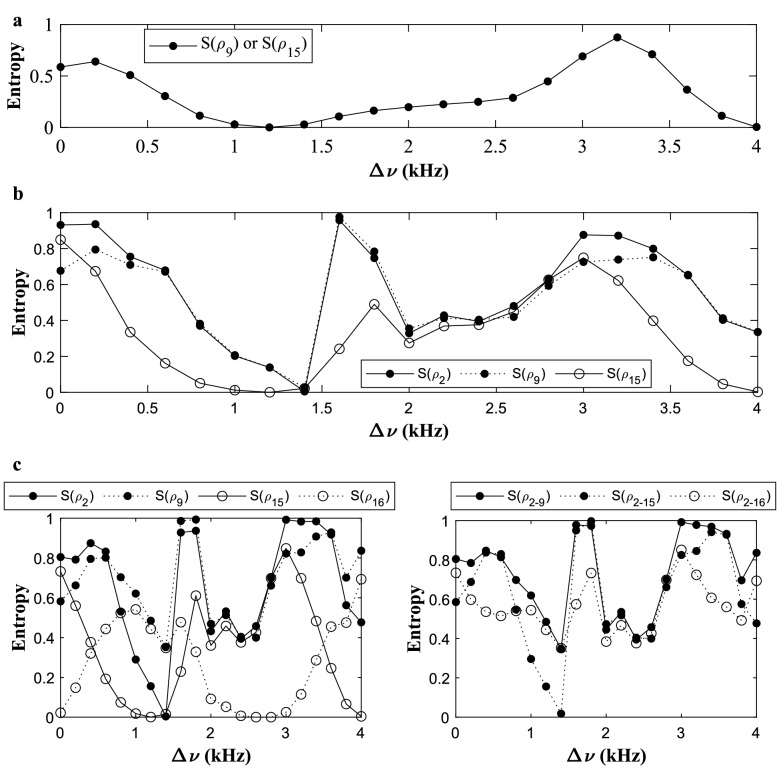
$N=\text{2,3},4$ entropy of entanglement. Variations in the entropy of entanglement $S\left(\rho \right)$ values of the partially traced states. The entropy is normalized to $\text{log}2$. (**a**) $S\left({\rho }_{9}\right)$ or $S\left({\rho }_{15}\right)$ of two logical phi-bits (9, 15). (**b**) $S\left({\rho }_{2}\right),S\left({\rho }_{9}\right),S\left({\rho }_{15}\right)$ of the three phi-bits (2, 9, 15). (**c**) Entropy of entanglement of the four phi-bits (2, 9, 15, 16) by taking the partial trace over one phi-bit i.e., $S\left({\rho }_{2}\right),S\left({\rho }_{9}\right),S\left({\rho }_{15}\right),S\left({\rho }_{16}\right)$ (left panel) or over two phi-bits i.e. $S\left({\rho }_{\text{2,9}}\right),S\left({\rho }_{\text{2,15}}\right),S\left({\rho }_{\text{2,16}}\right)$ (right panel).

**Figure 4:**
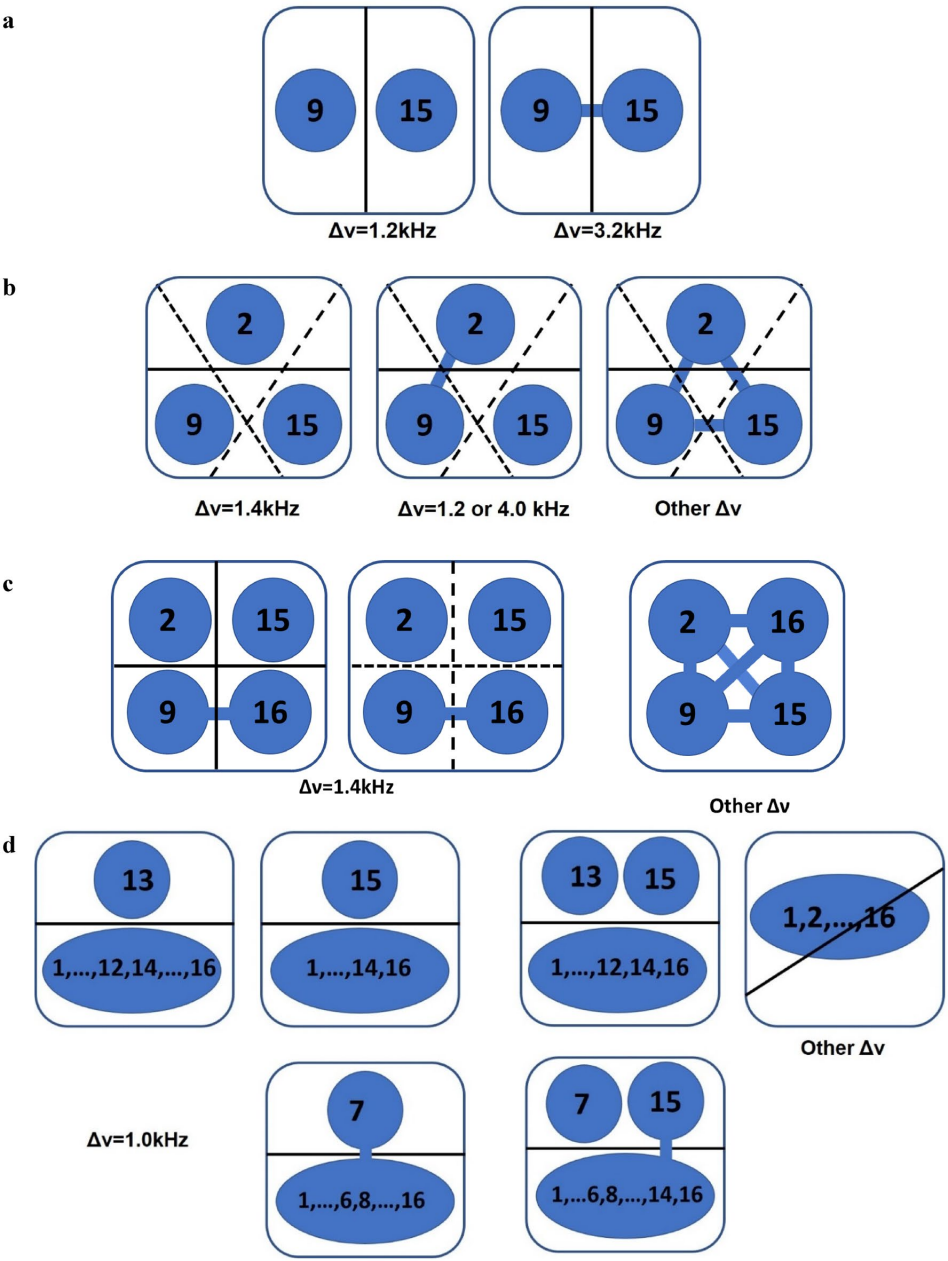
Illustration of nonseparability in $N=2, 3, 4, 16$ phi-bits. Composite systems illustrated as a blue square surrounding logical phi-bits depicted as numbered blue circles or ellipses. Nonseparability of phi-bit subsystems is illustrated by a blue link with partitioning illustrated by solid, short and long dashed black lines. A partitioning line intersecting a blue link represents a non-zero entropy of entanglement. (**a**) 1–1 partitioning of a *N* = 2 phi-bit system into two single phi-bits at the two detuning frequencies, Δν = 1.2 and 3.2 kHz. The first case is fully separable, the latter is inseparable. (**b**) Partitioning of *N* = 3 phi-bits into one phi-bit and a second subsystem containing two phi-bits. At Δν = 1.4 kHz, the entropy of entanglement for each type of 1–2 partition is almost zero and the three phi-bits are fully separable. At Δν = 1.2 or 4.0 kHz, separating phi-bit 15 from the other two leads to almost zero entropy, the long dashed black line does not intersect a blue link, a biseparable state. The other partitioning of the *N* = 3 system lead to non-zero entropies, phi-bits 2 and 9 are linked with the solid and short dashed black lines intersect the link. For most detuning frequencies, nonseparability is illustrated by intersection between solid, short and long dashed lines and blue links. (**c**) States of four phi-bits (2, 9, 15, 16) system; (**d**) *N* = 16 phi-bit system is separated into different partitions to show separability and nonseparability illustrated by solid black lines. Blue ellipses with numeral labels are used to illustrate composites of more than one phi-bit.

In Fig. 2a, we plot the experimental phase differences of the two logical phi-bits (9, 15), i.e., ${\varphi }_{12}^{(9)},{\varphi }_{12}^{(15)},{\varphi }_{13}^{(9)}$ and ${\varphi }_{13}^{(15)}$ for different values of the experimental detuning frequency parameter ${\Delta \upnu }$. In Fig. 2b, we plot the real and imaginary components of ${\psi }_{01}{\psi }_{10}$ and ${\psi }_{00}{\psi }_{11}$. We see that at $\Delta\upnu =1.2\text{ kHz or }4.0\text{ kHz}$, the state of the two logical phi-bits (9, 15) is separable, since both $\text{Real}\left({\psi }_{01}{\psi }_{10}\right)\approx \text{Real}\left({\psi }_{00}{\psi }_{11}\right)$ and $\text{Imag}\left({\psi }_{01}{\psi }_{10}\right)\approx \text{Imag}\left({\psi }_{00}{\psi }_{11}\right)$. For other detuning frequencies, the entropy of entanglement is non-zero indicating a nonseparable state, and at $\Delta\upnu = 3.2\text{ kHz}$ the state displays a large entanglement entropy. To further demonstrate nonseparability in this representation of the superposition of elastic states, we calculate the entropy of entanglement of subsystems in the composite system which is often non-zero. For this we determine the density matrix ($\rho =|{\psi }_{i}\rangle \langle {\psi }_{i}|$) and calculate the reduced density matrix associated with a partition of the system. The concept of reduced density matrix was first introduced by Dirac in 1930 and is calculated here by first taking the partial trace over one subsystem (one phi-bit) of the bipartite system constituted of the logical phi-bits pair (9, 15) i.e., either ${\rho }_{9}={\text{tr}}_{15}\left(\rho \right)$ or ${\rho }_{15}={\text{tr}}_{9}\left(\rho \right)$. We then proceed to characterize the classical “entanglement” between phi-bits pair (9, 15) through the calculation of a “entanglement” measure, such as von Neumann’s entropy of reduced states^11,12,36,37^, $S\left({\rho }_{9}\right)=-\text{tr}\left({\rho }_{9}\text{log}\left({\rho }_{9}\right)\right)=S\left({\rho }_{15}\right)=-\text{tr}\left({\rho }_{15}\text{log}\left({\rho }_{15}\right)\right)$. We also note that other entanglement measures, such as negativity or concurrence^38–40^ can also be used. In Fig. 3a, we plot the calculated entropy of partially traced states for different values of $\Delta \upsilon$. The entropy of entanglement is normalized to $\text{log}2$ i.e., ${S}_{9}(\rho )/\text{log}2$ (or ${S}_{15}(\rho )/\text{log}2$). This plot demonstrates that the entropy of entanglement varies over a very wide range of values from 0 to approaching 1 by merely tuning the parameter $\Delta \nu$. This result shows that we can explore a sizable portion of the Hilbert space defined by this phi-bit pair and we can experimentally access that space including nonseparable, as well as occasionally separable states. The contrast between separable and nonseparable states for this phi-bit pair is schematically illustrated in Fig. 4a for two values of $\Delta \nu$ (a detailed calculation of the entropy of entanglement values for those $\Delta \nu$ can be found in the Supplementary Note 3).

#### ${{N}}=3$

For a state with more than two phi-bits, the variety of “entangled” states is much richer. To characterize such states, we calculate the reduced density matrix and entropy of entanglement of the phi-bit triplet 2, 9, and 15. Their state is determined by the phase differences $\left({\varphi }_{12}^{(2)},{\varphi }_{13}^{(2)}\right), \left({\varphi }_{12}^{(9)},{\varphi }_{13}^{(9)}\right)$ and $\left({\varphi }_{12}^{(15)},{\varphi }_{13}^{(15)}\right)$. We first evaluate the reduced density matrix by tracing over either of the individual phi-bits $\left({\rho }_{2},{\rho }_{9},{\rho }_{15}\right)$ and we subsequently calculate the entanglement entropy of these partially traced states $\left(S\left({\rho }_{2}\right),S\left({\rho }_{9}\right),S\left({\rho }_{15}\right)\right)$ for different values of ${\Delta \upnu }$. These entropies are reported in Fig. 3b. For states composed of three subsystems, we predominantly find nonseparable states. Under special circumstances, there are several states either fully separable or biseparable. Since all the three entropy values of the partially traced states are almost zero at ${\Delta \upnu }=1.4\text{ kHz}$, the state vector corresponds to three logical phi-bits in a fully separable state i.e., ${\psi }_{\text{2,9},15}\approx {\psi }_{2}\otimes {\psi }_{9}\otimes {\psi }_{15}$. However, at different values of ${\Delta \upnu }$, either at ${\Delta \upnu }=1.2\text{ kHz}$ or ${\Delta \upnu }=4.0\text{ kHz}$, we see that only $S\left({\rho }_{15}\right)\approx 0$ and $S\left({\rho }_{2}\right)\ne 0\ne S\left({\rho }_{9}\right)$, which corresponds to a biseparable state that can be separated in a two phi-bit nonseparable state and a single separable phi-bit, i.e. ${\psi }_{\text{2,9},15}\approx {\psi }_{\text{2,9}}\otimes {\psi }_{15}$. The contrast between separable and nonseparable states for this phi-bit triplet is schematically illustrated in Fig. 4b for different values of the detuning parameter $\Delta \nu$.

#### ${{N}}=4$

In addition to finding separable, bi-separable and nonseparable states for the case of two and three phi-bit pairs (cf. Fig. 3b), it can be expected that for the case of four phi-bits the size of the Hilbert space and hence the variety of states would be even richer. Hence, we now focus on the composite system composed of four phi-bits (2, 9, 15, 16). We calculate the entropy of entanglement of the reduced density matrix by taking the partial trace over one subsystem composed of one phi-bit i.e., $S\left({\rho }_{2}\right),S\left({\rho }_{9}\right),S\left({\rho }_{15}\right),S\left({\rho }_{16}\right)$ (Fig. 3c left panel) and over a subsystem composed of two phi-bits i.e., $S\left({\rho }_{\text{2,9}}\right),S\left({\rho }_{\text{2,15}}\right),S\left({\rho }_{\text{2,16}}\right)$ (Fig. 3c right panel). Interestingly, this figure shows mostly nonseparable states which comprise the vast majority of the ${2}^{4}$ dimensional Hilbert space of the four phi-bit composite system. We note that for the detuning frequency of $\Delta \nu =1.4\text{ kHz}$, we see the unusual circumstance where $S\left({\rho }_{2}\right)$, $S\left({\rho }_{15}\right)$ (Fig. 3c left panel) and $S\left({\rho }_{\text{2,15}}\right)$ (Fig. 3c right panel) have entanglement entropies near zero. Therefore, at that detuning frequency we can write the state as: ${\psi }_{\text{2,9},\text{15,16}}={\psi }_{\text{2,15}}\otimes {\psi }_{\text{9,16}}={\psi }_{2}\otimes {\psi }_{15}\otimes {\psi }_{\text{9,16}}$. This state is naturally biseparable between pairs of phi-bits (2, 15) and (9, 16). It is clear simply from the entanglement entropies that the states that can be explored in our representation, defined by the choice of coefficients, is quite expansive even in the case of only 4 logical phi-bits and the limited number of detuning frequencies chosen.

For a conceptual interpretation of the results presented in Fig. 3c, we now depict the states of four phi-bits (2, 9, 15, 16) system in a graphic representation (Fig. 4c). In Fig. 4c, we partition between every individual phi-bit and the other three, and the partitioning is represented by solid lines making a right angle and cutting out a quadrant of the complete system. At Δν = 1.4 kHz, phi-bits 2 and 15 are separable from the rest of the system (Fig. 4c left panel). Separating phi-bit 9 or 16 from the remaining three leads to nonzero entropies of entanglement indicating that these two are nonseparable. This state is confirmed by partitioning into two halves (short and long dashed lines) (Fig. 4c middle panel). A non-zero entropy associated with the intersection between the long dashed line and the link between 9 and 16 confirms nonseparability. At any other detuning frequency, where the 1–3 and 2–2 partitioning give a non-zero entropy of entanglement, the system is nonseparable. Any partitioning will lead to an intersection of the black lines with blue links (Fig. 4c right panel).

#### ${{N}}=16$

Having established the insights that can be gained by examining the entanglement entropies for $N=2, 3, 4$ phi-bits, we now consider the ${2}^{16}$ dimensional Hilbert space defined by the 16 phi-bits listed in Table 1. The table displays the phi-bits from 1 to 16, ordered from low to high frequency and lists the minimum and maximum values of the calculated entanglement entropy and their corresponding detuning frequencies. The entropy of entanglement is calculated for a partitioning of the system into one of the phi-bits and another subsystem composed of the 15 other phi-bits (1–15 partition). Even at a glance, we notice wide variations in entanglement entropies with extrema at many different detuning frequencies for differing partitioning of the phi-bits, suggesting that we are accessing a large portion of the Hilbert space.

**Figure 5:**
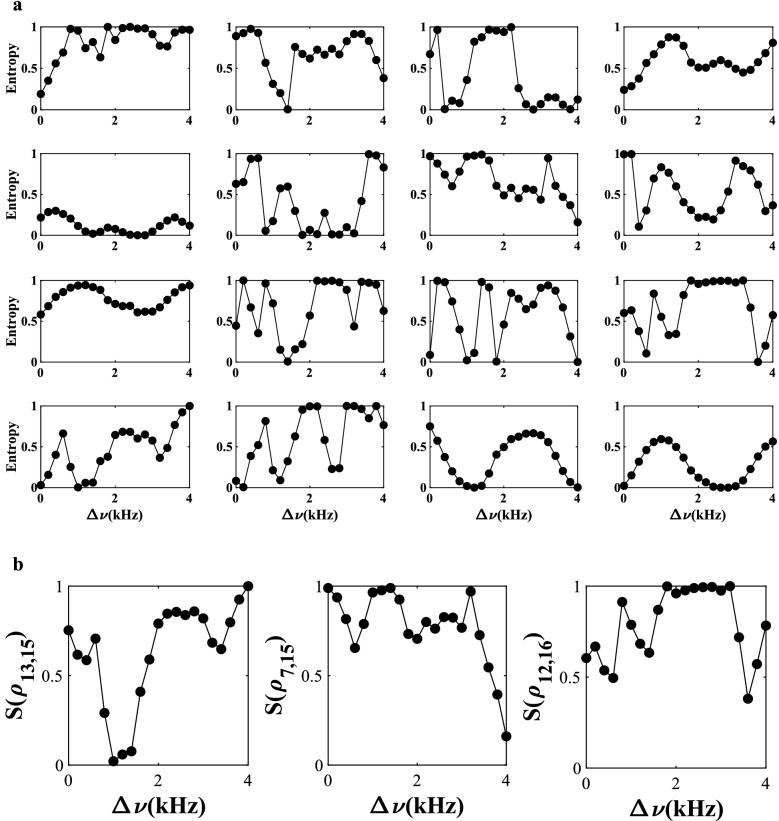
$N=16$ entropy of entanglement. Variations in the entropy of entanglement of the 16 phi-bits system. The entropy is normalized to $\text{log}2$. (**a**) 1–15 partition i.e., entropy of entanglement is calculated by taking the partial trace over one phi-bit subsystem $\left(S\left({\rho }_{1}\right),S\left({\rho }_{2}\right),\dots ,S\left({\rho }_{16}\right)\right)$. The entropy of entanglement of 1–15 phi-bit partitions are shown from top left (phi-bit 1) to bottom right (phi-bit 16). (**b**) 2–14 partition i.e., entropy of entanglement is calculated by taking the partial trace over two phi-bit subsystems $S\left({\rho }_{\text{13,15}}\right),S\left({\rho }_{\text{7,15}}\right),S\left({\rho }_{\text{12,16}}\right)$.

For an elastic state with sixteen phi-bits and the vast 2^16^ dimensional Hilbert space, the composite system can be partitioned in numerous ways and hence to study the entropy of entanglement values of all possible partial traced states is a formidable task. To illustrate the complexity of the nonseparable, and occasionally separable states, for 16 logical phi-bits system, we first calculate the entropy of entanglement values by taking the partial trace over all one phi-bit subsystems $\left(S\left({\rho }_{1}\right),S\left({\rho }_{2}\right),\dots ,S\left({\rho }_{16}\right)\right)$. The calculated entropy for different values of $\Delta \nu$ is reported in Fig. 5a. As is evident from Fig. 5a, for states composed of the sixteen subsystems the variety of entangled states is much richer and through the frequency tuning parameter, we can vary the entropy of entanglement values across the full spectrum of $0$ to $1$, in units of $\text{log}2$. Even though the Hilbert space is comprised mainly of nonseparable states, we can still find occasional separable phi-bits as is shown for the detuning frequency of $\Delta \nu =1.0\text{ kHz}$, where we see from Fig. 5a that $S\left({\rho }_{13}\right)\approx 0\approx S\left({\rho }_{15}\right)$. We can write ${\psi }_{1,\dots ,16}\approx {\psi }_{13}\otimes {\psi }_{1,\dots ,\text{12,14},\dots ,16}$ and ${\psi }_{1,\dots ,16}\approx {\psi }_{15}\otimes {\psi }_{1,\dots ,\text{14,16}}$. This is more evident if we calculate the entropy of entanglement value by taking the partial trace over two phi-bit subsystem (2–14 partition) (Fig. 5b left panel), for which we can write ${\psi }_{1,\dots ,16}\approx {\psi }_{\text{13,15}}\otimes {\psi }_{1,\dots ,\text{12,14,16}}$. On the other hand, since $S\left({\rho }_{7}\right)\ne 0$ at $\Delta \nu =1.0\text{ kHz}$ (Fig. 5a), we find the combined state to be nonseparable and we cannot write ${\psi }_{1,\dots ,16}\approx {\psi }_{\text{7,15}}\otimes {\psi }_{1,\dots ,\text{6,8},\dots ,\text{14,16}}$ (Fig. 5b middle panel) even though $S\left({\rho }_{15}\right)\approx 0$ at $\Delta \nu =1.0\text{ kHz}$.

A graphical illustration of nonseparability in the *N* = 16 phi-bit system is shown in Fig. 4d. Tracing out phi-bits 13 or 15 from the other 15 phi-bits at $\Delta \nu =1.0\text{ kHz}$ shows their separability (Fig. 4d top left panel). We expect that partitioning both phi-bits 13 and 15 from the other 14 phi-bits at this detuning frequency will also be separable as is demonstrated in Fig. 5 and illustrated in Fig. 4d. The entropy of entanglement obtained by tracing out phi-bit 7 shows that it is nonseparable from the other 15 phi-bits (cf. Fig. 4d bottom left panel).) and hence we again find a nonseparable state by tracing out phi-bits 7 and 15 from the others (Fig. 4d bottom right panel). Detuning conditions for which none of the individual phi-bits can be separated from the other 15 suggests states that are non-separable (Fig. 4b top right panel). This non-separability would be occurring for any type of partitioning.

The complexity of the 16 phi-bit representation of the 2^16^ dimensional Hilbert space would allow for many other ways of partitioning the phi-bits into groups, most of these partitions leading to nonseparable states that are the signature of classical entanglement.

## Discussion

We have experimentally demonstrated classical entanglement, i.e., nonseparability, for acoustic logical phi-bits resulting from the partitioning in the spectral domain of the acoustic field of an externally driven array of three acoustic waveguides elastically coupled along their length. Each phi-bit is a two-level nonlinear mode of vibration whose state is characterized by a frequency and two independent relative phases between waveguides. Multi phi-bit systems are analogous to qubit systems in the sense that their representation can be endowed with a tensor product structure scaling exponentially with the number, $N$, of bits. The states of a composite system of $N$ phi-bits can then be represented and manipulated in a ${2}^{N}$ dimensional Hilbert space. The nonseparability of multiple phi-bit superpositions of states has been characterized by calculating the entropy of entanglement of various partitions of four representative multi phi-bit composite systems, namely, $N=2, 3, 4,$ and $16$. By partitioning the smaller composite systems, i.e., partially tracing the density matrix used to calculate the entropy of entanglement, we have shown the existence of states with different degree of classical entanglement ranging from separable, to biseparable, to nonseparable. Most remarkably, the entropy of entanglement of a 16 phi-bit system partitioned in several ways (i.e., tracing out of the density matrix one phi-bit or pairs of phi-bits) indicates that we can realize large scale entangled states. Detuning the drivers’ frequency of the externally driven classical acoustic system, is one possible way to easily navigate a substantial portion of the Hilbert space of multi phi-bit representations. This simple approach offers access to the 2^16^ dimensional Hilbert space spanning much of its complex nonseparable state volume. We should note that it is not our aim to simulate quantum systems nor to establish a complete analogy with them. Several features still distinguish composite phi-bit systems from true quantum systems, namely locality vs quantum nonlocality, superpositions of states involving amplitudes vs probability amplitudes. The logical phi-bits are defined in the spectral domain enabling their spatial co-location thus doing away with any issue of distance in their interactions. Multi phi-bit superpositions of states are directly measurable by contact (e.g., transducers), or non-contact methods (e.g., laser ultrasonics). Composite phi-bit systems are stable against decoherence and do not suffer from wave function collapse upon measurement. The experimental results reported here and the analogy between classical and quantum entanglement within the framework of nonseparability suggest the possibility of harnessing the complexity of classical entanglement to challenge the current viewpoint on the superiority of quantum systems in meeting the needs of future information science.

## Methods

The experimental realization of nonlinear acoustic waveguide-transducer-amplifier-generator platform consists of three aluminum rods (McMaster-Carr multipurpose 6061 aluminum rod with certification 1/2′′ diameter, 0.609 m length, and density $\rho =2660$ $\text{kg}/{\text{m}}^{3}$). The lateral gap between the rods is filled with epoxy (50,176 KwikWeld Syringe). Two sets of transducers (V133-RM—Olympus IMS) are used to drive and to detect the acoustic field at the ends of the rods. The two driving transducers are connected to waveform generators (B&K Precision 4055B) through PD200 amplifiers (PD200 is a high bandwidth, low-noise linear amplifier). The three recording transducers are connected to Tektronix oscilloscope (MDO3024) to detect signals at the rod end. The input (driving) and output (response) signals are collected in the oscilloscope. The waveform generators are connected to a computer to control the experiment, and to perform data processing the oscilloscopes are also connected to a digital computer.

## Supplementary Information

Supplementary Information.

## Data Availability

The data that support our findings of the present study are available from the corresponding author upon reasonable request.
