# Supplementary material for: Experimental classical entanglement in a 16 acoustic qubit-analogue
Source: Sci Rep. 2021 Dec 20;11:24248. doi: 10.1038/s41598-021-03789-5 (PMC8688442; doi:10.1038/s41598-021-03789-5)
Supplement: Supplementary file 1 — Supplementary Information. [file 41598_2021_3789_MOESM1_ESM.pdf]

## SUPPLEMENTARY INFORMATION

### Supplementary Note 1. Nonlinearly coupled array of elastic waveguides

We consider three one-dimensional elastic waveguides coupled elastically along their length. The system is stimulated externally at some position $x = 0$ (Fig. S1) by piezoelectric transducers at each end of the waveguides electrically driven through signal generators and amplifiers.

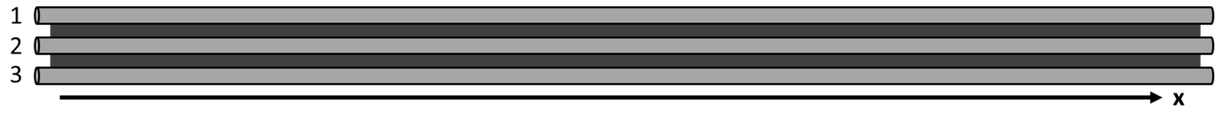


**Fig. S1| Coupled Waveguides.** Schematic of the parallel arrays of coupled three waveguides.

In the long wavelength limit the wave equation takes the form:

$$\left[\left(\frac{\partial^2}{\partial t^2} - \beta^2 \frac{\partial^2}{\partial x^2} + \mu \frac{\partial}{\partial t}\right)\overleftrightarrow{I} + \alpha^2 \overleftrightarrow{M}\right]\vec{U} + \varepsilon \vec{G}(\vec{U}) = \vec{F}_1 \delta_{x=0} \cos \omega_1 t + \vec{F}_2 \delta_{x=0} \cos \omega_2 t \qquad (1)$$

The parameter $\beta$ is proportional to the speed of sound in the waveguides. $x$ represents the position along the waveguides. $\mu$ is a damping parameter. $\overleftrightarrow{I}$ is the identity matrix. $\alpha$ stands for the coupling strength of the epoxy between waveguides with $\overleftrightarrow{M}$ being the matrix describing the elastic coupling between the 3 waveguides. $\vec{F}_1$ and $\vec{F}_2$ are $3\times 1$ vectors representing the driving forces applied on each waveguide for two different driving angular frequencies $\omega_1 = 2\pi f_1$ and $\omega_2 = 2\pi f_2$. For the sake of mathematical simplicity, in this example, we reduce the expression of the driving forces to the positive frequencies $\vec{F}_1 \delta_{x=0} e^{i\omega_1 t} + \vec{F}_2 \delta_{x=0} e^{i\omega_2 t}$ and therefore we limit nonlinear effects to the mixing of these positive frequencies.

In the case of a planar array of waveguides, the coupling matrix takes the form:

$$\overleftrightarrow{M} = \begin{pmatrix} 1 & -1 & 0 \\ -1 & 2 & -1 \\ 0 & -1 & 1 \end{pmatrix} \tag{2}$$

The $3 \times 1$ vector $\vec{U} = (U_1, U_2, U_3)$ represents the displacement in waveguides 1, 2 and 3. The term $\varepsilon\vec{G}(\vec{U})$ stands for the nonlinearity of the system composed of the waveguides, transducers, amplifiers and signal generators. $\varepsilon$ is a measure of the strength of the nonlinear term. The physical origin of the nonlinearity, that is, mechanical or electrical, is not critical as long as the nonlinearity depends on displacement $\vec{U}$. The nonlinear term can arise from intrinsic or geometric nonlinearity of the elastic system. It may also result from nonlinear feedback between the amplifiers/generators through the coupled transducers. A key feature is that in both cases the nonlinear term would be dependent on the elastic displacement field and therefore would be able to mix waves with different frequencies. For the sake of illustration, we consider here a simple quadratic nonlinearity which depends on the difference on the displacement between adjacent waveguides:

$$\vec{G}(\vec{U}) = \begin{pmatrix} (U_1 - U_2)^2 \\ -(U_1 - U_2)^2 + (U_2 - U_3)^2 \\ -(U_2 - U_3)^2 \end{pmatrix} \tag{3}$$

We solve Eq. (1) within perturbation theory. Assuming a small $\varepsilon$, we expand the displacement field to first order in perturbation:

$$\vec{U} = \vec{U}^{(0)} + \varepsilon\vec{U}^{(1)} \tag{4}$$

Inserting Eq. (4) into Eq. (1) yields to zeroth order in perturbation:

$$\left[\left(\frac{\partial^2}{\partial t^2} - \beta^2\frac{\partial^2}{\partial x^2} + \mu\frac{\partial}{\partial t}\right)\overleftrightarrow{I} + \alpha^2\overleftrightarrow{M}\right]\vec{U}^{(0)} = \vec{F}_1\delta_{x=0}e^{i\omega_1 t} + \vec{F}_2\delta_{x=0}e^{i\omega_2 t} \tag{5}$$

We now define $\lambda_n$ and $\vec{E}_n$ with $n$=1, 2, 3, the eigen values and eigen vectors of the matrix $\overleftrightarrow{M}$. The $\vec{E}_n$ represent the spatial eigen modes across the waveguides with components $E_{n,j}$, $j = 1,2,3$. One of the eigen vector for which $\lambda_1 = 0$, namely $\vec{E}_1^T = \frac{1}{\sqrt{3}}(1,1,1)$, does not involve transfer of energy between the waveguides via the coupling. This trivial case is equivalent to three independent waveguides. The other two eigen modes of the coupling matrix with eigen values $\lambda_2 = 1$, and $\lambda_3 = 3$, are:

$$\vec{E}_2 = \begin{pmatrix} E_{2,1} \\ E_{2,2} \\ E_{2,3} \end{pmatrix} = \frac{1}{\sqrt{2}}\begin{pmatrix} 1 \\ 0 \\ -1 \end{pmatrix}, \vec{E}_3 = \begin{pmatrix} E_{3,1} \\ E_{3,2} \\ E_{3,3} \end{pmatrix} = \frac{1}{\sqrt{6}}\begin{pmatrix} 1 \\ -2 \\ 1 \end{pmatrix}$$

We can write:

$$\overleftrightarrow{M}\vec{E}_n = \lambda_n \overleftrightarrow{I}\vec{E}_n \tag{6}$$

Since the $\vec{E}_n$ form a complete orthonormal basis, we can write the displacement vector as:

$$\vec{U}^{(0)} = \sum_n u_n^{(0)} \vec{E}_n \tag{7}$$

Since Eq. (5) is linear, we focus on a single driving frequency, $\vec{F}_l$ with $l$=1 or 2. That is we seek solutions of the equation:

$$\left[\left(\frac{\partial^2}{\partial t^2} - \beta^2 \frac{\partial^2}{\partial x^2} + \mu \frac{\partial}{\partial t}\right)\overleftrightarrow{I} + \alpha^2 \overleftrightarrow{M}\right]\vec{U}_l^{(0)} = \vec{F}_l \delta_{x=0} e^{i\omega_l t} \tag{8}$$

The $3 \times 1$ vector, $\vec{F}_l$, is also expressed in the $\vec{E}_n$ basis:

$$\vec{F}_l = \sum_n F_n^{(l)} \vec{E}_n \tag{9}$$

The $F_n^{(l)}$'s are therefore defined as the dot product $\vec{F}_l.\vec{E}_n$ between the two $3 \times 1$ vectors. Inserting Eqs (6), (7) and (8) in Eq. (1) leads to a set of 3 equations of the form:

$$\left(\frac{\partial^2}{\partial t^2} - \beta^2 \frac{\partial^2}{\partial x^2} + \mu \frac{\partial}{\partial t} + \alpha^2 \lambda_n\right) u_{n,l}^{(0)} = F_n^{(l)} \delta_{x=0} e^{i\omega_l t} \quad (10)$$

We seek plane wave solutions which follow the driver in time:

$$u_{n,l}^{(0)} = \sum_{k_n} A_{n,l}(k_n)\, e^{ik_n x} e^{i\omega_l t} \quad (11)$$

In Eq. (11) the summation over the wave numbers, $k_n$, is discrete, thus implying that the waveguides have the same finite length.

Inserting Eq. (11) into Eq. (10) evaluated at $x = 0$ yields the driven complex amplitudes:

$$A_{n,l}(k_n) = \frac{F_n^{(l)}}{\omega_{0,n}^2(k_n) - \omega_d^2 + i\mu\omega} \quad (12)$$

where the characteristic frequency

$$\omega_{0,n}^2(k_n) = \beta^2 k_n^2 + \alpha^2 \lambda_n \quad (13)$$

The displacement field is therefore obtained as

$$\vec{U}_l^{(0)} = \sum_{n=1}^{3} \vec{E}_n \sum_{k_n} A_{n,l}(k_n)\, e^{ik_n x} e^{i\omega_l t} \quad (14)$$

with the complex resonant amplitudes given by Eq. (12). When driving the system at two different frequencies, the complete displacement field is:

$$\vec{U}^{(0)} = \vec{U}_1^{(0)} + \vec{U}_2^{(0)} = \sum_{n=1}^{3} \vec{E}_n \left(\sum_{k_n} A_{n,1}(k_n)\, e^{ik_n x} e^{i\omega_1 t} + \sum_{k'_n} A_{n,2}(k'_n)\, e^{ik'_n x} e^{i\omega_2 t}\right) \quad (15)$$

Note that in Eq. (16) we have used two independent summation indices for the wavenumber, namely $k_n$ and $k'_n$.

To first order in perturbation, the equation of motion takes the form:

$$\left[\left(\frac{\partial^2}{\partial t^2} - \beta^2 \frac{\partial^2}{\partial x^2} + \mu \frac{\partial}{\partial t}\right)\overleftrightarrow{I} + \alpha^2 \overleftrightarrow{M}\right] \vec{U}^{(1)} + \vec{G}(\vec{U}^{(0)}) = 0 \quad (16)$$

In that equation, the zeroth order displacement drives the system with mixed frequencies.

Let us define $S_{n,l} = \sum_{k_n} A_{n,l}(k_n)\, e^{ik_n x}$, we calculate the first component of the nonlinear term as:

$$\left\{\left(U_1^{(0)} - U_2^{(0)}\right)^2\right\}_{\omega_1,\omega_2} = \left(\sum_{n=1}^{3}(E_{n,1} - E_{n,2})\left(S_{n,l=1}e^{i\omega_1 t} + S_{n,l=2}e^{i\omega_2 t}\right)\right)^2 \quad (17)$$

Focusing on the terms which correspond to mixed frequencies only, we reduce equation (17) to

$$\left(U_1^{(0)} - U_2^{(0)}\right)^2 = \sum_{n=1}^{3}\sum_{m=1}^{3} g_{n,m}^{1,2} S_{n,l=1} S_{m,l=2}\, e^{i(\omega_1+\omega_2)t} \quad (18)$$

where $g_{n,m}^{1,2} = 2(E_{n,1} - E_{n,2})(E_{m,1} - E_{m,2})$.

The contribution of each of the nine $\{n,m\}$ terms to $\vec{G}(\vec{U}^{(0)})$ in Eq. (18) is $\begin{pmatrix} g_{n,m}^{1,2} \\ -g_{n,m}^{1,2} + g_{n,m}^{2,3} \\ -g_{n,m}^{2,3} \end{pmatrix}$.

Each of these $3 \times 1$vectors can be expended on the complete basis of the $\vec{E}_n$ in the form $\sum_p \tilde{g}_p^{(n,m)} \vec{E}_p$.

The nonlinear term is then expressed as:

$$\vec{G}\left(\vec{U}^{(0)}\right) = \sum_p \sum_{n=1}^{3}\sum_{m=1}^{3} \tilde{g}_p^{(n,m)} \vec{E}_p S_{n,l=1} S_{m,l=2}\, e^{i(\omega_1+\omega_2)t} \quad (20)$$

Similarly, we expand the first order displacement field for each $\{n,m\}$ term on the same basis: $\vec{U}^{(1)}(n,m) = \sum_p u_p^{(1)}(n,m)\vec{E}_p$. With this we write:

$$\vec{U}^{(1)} = \sum_p \sum_{n=1}^{3}\sum_{m=1}^{3} u_p^{(1)}(n,m)\vec{E}_p S_{n,l=1} S_{m,l=2}\, e^{i(\omega_1+\omega_2)t} \quad (21)$$

Inserting these expansions in Eq. (16), the equations of motion to first order in perturbation reduce to three equations with $p$=1, 2, 3:

$$\left(-i(\omega_1+\omega_2)^2+\beta^2(k_n+k_m)^2+i\mu(\omega_1+\omega_2)+\alpha^2\lambda_p\right)u_p^{(1)}(n,m)+\tilde{g}_p^{(n,m)}=0 \tag{22}$$

leading to the resonant amplitude

$$u_p^{(1)}(n,m)=\frac{-\tilde{g}_p^{(n,m)}}{\left(-(\omega_1+\omega_2)^2+\beta^2(k_n+k_m)^2+i\mu(\omega_1+\omega_2)+\alpha^2\lambda_p\right)} \tag{23}$$

Evaluating the complete first order displacement field for a given $\omega_1+\omega_2$ at one end of the waveguide array (say $x$=0), gives

$$\vec{U}^{(1)}=\sum_p\sum_{n=1}^3\sum_{m=1}^3 u_p^{(1)}(n,m)\vec{E}_p\sum_{k_n}\sum_{k_m}A_{n,1}(k_n)\,A_{m,2}(k_m)\,e^{i(\omega_1+\omega_2)t} \tag{24}$$

This displacement field can be expressed in the compact form:

$$\vec{U}^{(1)}=\begin{pmatrix}C_1e^{i\varphi_1}\\C_2e^{i\varphi_2}\\C_3e^{i\varphi_3}\end{pmatrix}e^{i(\omega_1+\omega_2)t} \tag{25}$$

The complex resonant amplitudes, $u_p^{(1)}(n,m)$, $A_{n,1}(k_n)$ and $A_{m,2}(k_m)$ introduce a phase difference between the components of the displacement field $\vec{U}^{(1)}$. Setting one of these phase differences to zero, the other two phase differences form the experimental measurables:

$$\vec{U}^{(1)}=\begin{pmatrix}1\\\hat{C}_2e^{i\varphi_{12}}\\\hat{C}_3e^{i\varphi_{13}}\end{pmatrix}e^{i(\omega_1+\omega_2)t} \tag{26}$$

Where $\hat{C}_2$ and $\hat{C}_3$ are normalized to $C_1$ and $\varphi_{12}=\varphi_2-\varphi_1$ and $\varphi_{13}=\varphi_3-\varphi_1$. The argument made here was illustrated for a simple case of quadratic nonlinearity. Here, we have also limited in the discussion to the case of positive frequencies. However, the cosine functions in Eq. (1) also

contain complex exponentials with negative terms, namely $e^{-i\omega_1 t}$ and $e^{-i\omega_1 t}$. Nonlinear mixing of positive and negative driving frequencies will lead to first order displacements in $e^{\pm i(\omega_1-\omega_2)t}$.

**Supplementary Note 2. Representations of multi phi-bit systems**

In Supplementary Note 1, we have shown that the nonlinear displacement field to first order in perturbation measured at one end of the externally driven array of three coupled elastic waveguides can be represented as (Eq. (26)):

$$\vec{U}_{(i)}^{(1)} = \begin{pmatrix} 1 \\ \hat{C}_2 e^{i\varphi_{12}} \\ \hat{C}_3 e^{i\varphi_{13}} \end{pmatrix} e^{i\omega^{(i)}t} \tag{27}$$

where the amplitudes of the field at the end of waveguides 2 and 3, $\hat{C}_2$ and $\hat{C}_3$ are normalized to the amplitude at the end of waveguide 1, $C_1$. Here $\varphi_{12} = \varphi_2 - \varphi_1$ and $\varphi_{13} = \varphi_3 - \varphi_1$, are the phases measured at the end of waveguides 2 and 3 relative to that of rod 1. We note that the phase differences $\varphi_{12}$ and $\varphi_{13}$ depend on "$i$" and should therefore be formulated as $\varphi_{12}^{(i)}$ and $\varphi_{13}^{(i)}$. However, for the sake of simplifying the notation, we drop the superscript ($i$). $\omega^{(i)} = (\omega_1 + \omega_2)$, is the frequency of a nonlinear mode, "$i$", mixing the two drivers' frequencies $\omega_1$ and $\omega_2$. Other modes result from different combinations of the driving frequencies but the general form of Eq. (27) is retained. We can reduce this description by dropping direct reference to rod 1 in the representation. In that case, we define the measured field at the end of the waveguides by a $2 \times 1$ vector:

$$\vec{U}_{(i)} = \begin{pmatrix} \hat{c}_2 e^{i\varphi_{12}} \\ \hat{c}_3 e^{i\varphi_{13}} \end{pmatrix} e^{i\omega^{(i)}t} \tag{28}$$

$\hat{c}_2$ and $\hat{c}_3$ are obtained by normalizing the $2 \times 1$ vector. Each nonlinear mode, "$i$" is equivalent to an oscillator with characteristic frequency $\omega^{(i)}$ which normalized phase dependent amplitude is characterized by a $2 \times 1$ vector. This is the representation of one phi-bit. An equation of motion representing the evolution of oscillations along the positive timeline of this oscillator may take the form[S1]:

$$\left[\left(-\frac{d}{dt} + i\omega^{(i)}\right)\overleftrightarrow{I}_{2\times2} + \overleftrightarrow{C}_{2\times2}^{(i)}\right]\vec{V}_{(i)} = 0 \quad (29)$$

Seeking solutions in the general form $\vec{V}_{(i)} = \begin{pmatrix} V_1^{(i)} \\ V_2^{(i)} \end{pmatrix} e^{iw^{(i)}t}$, one recovers the representation given by Eq. (28) when $\overleftrightarrow{C}_{2\times2}^{(i)} = \begin{pmatrix} -1 & X \\ -X^{-1} & 1 \end{pmatrix}$ with $X = \hat{c}_2 e^{i\varphi_{12}} / \hat{c}_3 e^{i\varphi_{13}}$. The eigen values of the matrix $\overleftrightarrow{C}_{2\times2}^{(i)}$ are equal to zero. Its Eigen vectors are the $2 \times 1$ vectors of Eq. (28). The eigen frequency $w^{(i)}$ is equal to the characteristic frequency of the oscillator, $\omega^{(i)}$. The product of functions $e^{iw^{(i)}t}$ and the eigen vectors of $\overleftrightarrow{C}_{2\times2}^{(i)}$ form a complete basis for the states of the oscillator, "$i$". These basis vectors define the Hilbert space, $h_{(i)}$, of a single oscillator "$i$".

We can now construct equations of motion for a multipartite system composed of *N* independent phi-bits:

$$\left[\left(-\frac{d}{dt} + i\omega^{(1)} + \cdots + i\omega^{(N)}\right)\overleftrightarrow{I}_{2^N\times2^N} + \overleftrightarrow{C}_{2\times2}^{(1)} \otimes \ldots \otimes \overleftrightarrow{C}_{2\times2}^{(N)}\right]\vec{W} = 0 \quad (30)$$

The solutions of these equations are tensor products of single phi-bit states, namely: $\vec{W} = \vec{U}_{(1)} \otimes \ldots \otimes \vec{U}_{(N)}$. The tensor product of the basis vectors of single phi-bit forms a complete basis for the states of the non-interacting multipartite system. This basis defines a $2^N$ dimensional Hilbert space, *H*, which is the tensor product of the *N* Hilbert spaces of the individual oscillators, $H = h_{(1)} \otimes$

$\ldots \otimes h_{(N)}$. In the case of interacting phi-bits, the tensor product $\overleftrightarrow{C}_{2\times 2}^{(1)} \otimes \ldots \otimes \overleftrightarrow{C}_{2\times 2}^{(N)}$ in Eq. (30) may be replaced by a general matrix $\overleftrightarrow{C}_{2^N \times 2^N}$. The Hilbert space spanned by the states of the interacting system is the same as that of the non-interacting system. A state of the interacting system may then be written as a linear combination (with complex coefficients) of the basis vectors of *H*. We can then define new representations of the *N* phi-bit system by applying a unitary transformation to the basis of *H*. In this study, we have considered a transformation such that the complex coefficients of a state of the multipartite system take the form: $1 + e^{i\sum_{j=1}^{N} \varphi_{1q_j}^{(j)}}$ where $q_j = 2,3$. Here, we have brought back the superscript (*j*) to differentiate the phi-bits. This is but one of the many possible representations of the *N* phi-bit system.

**Supplementary Note 3. Illustrative examples of separable and non-separable states in *N*=2 phi-bit system**

In a two logical phi-bits (phi-bits 9 and15) bipartite system, for the particular tuning parameter $\Delta\nu = 1.2k\text{Hz}$, the experimentally measured elastic state is represented by the vector:

$$|\psi\rangle = \begin{pmatrix} 0.0878 + 0.2510i \\ 0.0945 + 0.2592i \\ 0.5348 - 0.3803i \\ 0.5248 - 0.3836i \end{pmatrix},$$

and the corresponding density matrix $\rho = |\psi\rangle\langle\psi|$. The corresponding reduced density matrix are calculated as:

$$\rho_9 = \begin{pmatrix} 0.5013 & 0.4999 - 0.0065i \\ 0.4999 + 0.0065i & 0.4987 \end{pmatrix},$$

$$\rho_{15} = \begin{pmatrix} 0.1468 & -0.0983 - 0.3399i \\ -0.0983 + 0.3399i & 0.8532 \end{pmatrix}.$$

These reduced density matrices give values of the entropy of entanglement which are almost zero which indicates that this state is separable. Indeed the state vector can be written as:

$$|\psi\rangle = \begin{pmatrix} 0.0878 + 0.2510i \\ 0.0945 + 0.2592i \\ 0.5348 - 0.3803i \\ 0.5248 - 0.3836i \end{pmatrix} \approx \begin{pmatrix} 0.1235 + 0.3824i \\ 0.8217 - 0.5549i \end{pmatrix} \otimes \begin{pmatrix} 0.6616 - 0.0160i \\ 0.6861 - 0.0256i \end{pmatrix}$$

$$\approx [(0.1235 + 0.3824i)|0\rangle_9 + (0.8217 - 0.5549i)|1\rangle_9]$$

$$\otimes [(0.6616 - 0.0160i)|0\rangle_{15} + (0.6861 - 0.0256i)|1\rangle_{15}] .$$

For a different detuning parameter $\Delta\nu = 3.2k$Hz, the elastic state is represented by the state vector

$$|\psi\rangle = \begin{pmatrix} 0.0201 - 0.1363i \\ 0.2932 + 0.4366i \\ 0.6779 - 0.4242i \\ 0.0689 - 0.2455i \end{pmatrix},$$

and the corresponding reduced density matrix:

$$\rho_9 = \begin{pmatrix} 0.6584 & 0.0973 - 0.0884i \\ 0.0973 + 0.0884i & 0.3416 \end{pmatrix},$$

$$\rho_{15} = \begin{pmatrix} 0.2955 & -0.0155 - 0.0182i \\ -0.0155 + 0.0182i & 0.7045 \end{pmatrix},$$

This results in entropy value of $0.874 \log 2$ which indicates that the state corresponding to a detuning frequency $\Delta\nu = 3.2k$Hz is nonseparable. The state vector cannot be written as a tensor product of two vectors.

**Supplementary References**

S1. Deymier, P. & Runge, K. *Sound Topology, Duality, Coherence and Wave-Mixing: An Introduction to the Emerging New Science of Sound*. (Springer International Publishing, 2017).
